# Test Paper for Colorimetric Inspection of Fatty Acids and Edible Oils

**DOI:** 10.3390/s18103252

**Published:** 2018-09-27

**Authors:** Feng Zhang, Xiaojie Wang, Xu Jie, Weili Wei

**Affiliations:** School of Pharmaceutical Sciences, Chongqing University, Chongqing 401331, China; zhang_feng@cqu.edu.cn (F.Z.); 20141802037@cqu.edu.cn (X.W.); jiexu@cqu.edu.cn (X.J.)

**Keywords:** test paper, colorimetric sensor, fatty acid, edible oil

## Abstract

Fatty acids (FAs) are of interest to the areas of food science and medicine because they are important dietary sources of fuel for animals and play important roles in many biological processes. The health effects of FAs are different due to the diversity of olefinic bonds in the alkyl chains including number, position and configuration. However, the discrimination of FAs is difficult from a chemical sensing perspective due to the lack of diversity in terms of functional groups. Until now, only a few chemosensors have been developed for selective sensing of FAs based on their overall shape, however they are still limited in discrimination of FAs with subtle structural differences, moreover, they cannot be used for rapid and in situ inspections. Herein, for the first time, we designed a test paper for in situ colorimetric inspection for FAs based on the combination of the highly selective binding of Ag^+^ to olefinic bonds and Ag^+^ mediated color variation of 3,3′,5,5′,-tetramethylbenzidine. As a result, the sensor exhibited high sensitivity and good selectivity for five FAs with subtle structural differences. Furthermore, our method described herein was successfully applied to monitor the structural variations of FAs and quality changes in mixture edible hot pot oils with heat treatment in time course. Hence, the test paper presented herein holds great potential in the inspection of fats and edible oils in food industries.

## 1. Introduction

Fatty acids (FAs) are the primary components of animal fats and vegetable oils, which are important dietary sources of fuel and are required for energy storage, membrane proliferation, and the generation of signaling molecules [1]. FAs are aliphatic monocarboxylic acids with carbon atoms ranging from 4 to 28, which are obtained by the hydrolysis of natural triglycerides, phospholipids, and cholesterol esters. According to the number of their olefinic bond, they are classified into three categories, including saturated fatty acids (SFAs) with no olefinic bond, mono-unsaturated fatty acids (mono-UFAs) with one olefinic bond and poly-unsaturated fatty acids (poly-UFAs) with more than one olefinic bond [2]. In nature, UFAs generally have *cis* configurations, the opposed *trans*-UFAs (TUFAs) naturally present in ruminant adipose tissue and related dairy products. In food production, liquid *cis*-UFA (CUFA) esters such as vegetable oils are hydrogenated or partially hydrogenated to produce SFAs or TUFAs, which are more stable and easier to store and use [3]. Thus, the SFAs and TUFAs are widely produced and used in the food industry such as for the production of margarine, snack foods, baked foods and frying fast foods in restaurants [4]. However, extensive experimental and observational data indicate that excessive use of SFAs and TUFAs can easily lead to cardiovascular and related diseases [5,6,7]. According to world health organization (WHO) estimates, the intake of TUFAs leads to more than 500,000 deaths of people from cardiovascular related diseases every year [8]. In order to protect human health, WHO enacted a plan named REPLACE in 14 May 2018, which is a stipulation to eliminate the industrially-produced TUFAs in the global food industry [8]. Therefore, the approaches to determine FAs profile in food industries are important in order to correct nutrition labeling and control of labeling authenticity, which is of great significance to the implementation of the plan of REPLACE.

The structures of FAs are diversity, because the length (carbon number) and unsaturation degree (olefinic bond number) of alkyl chains, position and configuration (*cis*/*trans*) of the olefinic bond in FAs are different. The chemical discrimination of FAs is difficult, because they lack special functional groups other than the hydrophobic alkyl chain and carboxyl group. Currently, the most used methods for the determination of FAs are based on gas chromatography (GC) or liquid chromatography (LC) coupling with mass spectrometry (MS) analysis [9,10,11]. However, the GC/LC-MS techniques suffer from some drawbacks, such as being time-consuming, expensive, operationally complex and relying on sophisticated instrumentation that cannot be used for rapid and in situ analysis. Moreover, a variety of regio- and stereoisomers can exist for FAs, posing a challenge for MS based analyses. Chemosensors have been widely used in various fields because of their rapid, facile and convenient features, and these features just make up for the inadequacies of the GC/LC-MS related techniques. Until now, only a few colorimetric and fluorimetric chemosensors have been developed for selective sensing of FAs based on their overall shape [12,13]. The main capability of these chemosensors is discrimination of mono-CUFAs (with bent alkyl chain) from mono-TUFAs and SFAs (with linear alkyl chains), but they do not show promise in differentiating poly-UFAs and SFAs. Therefore, a portable sensing method for in situ inspection of FAs with different degree of unsaturation, position of olefins, and the configuration of the olefins is urgent but difficult.

It is well known that silver ion (Ag^+^) can complex with olefinic bonds reversibly [14]. The stabilities of Ag^+^-olefin complexes are highly dependent on the properties of olefins that can usually be divided into two groups, namely steric (chain length, *cis*/*trans* isomers and number of olefinic bonds) and electronic (conjugation and electron cloud density) properties [15]. Thus, Ag^+^ chromatography has been widely used for the separation of unsaturated compounds with subtle structural differences such as FAs and sterols [16,17,18,19]. However, a FAs sensor based on the selective complexation between Ag^+^ and olefinic bonds has never been explored. Interestingly, the colorless 3,3′,5,5′,-tetramethylbenzidine (TMB) can be oxidized by Ag^+^ and turn blue, and the color intensity is positively correlated with the concentration of Ag^+^ [20,21,22]. When combining the highly selective binding ability of Ag^+^ to olefinic bonds and Ag^+^ mediated color variation of TMB, a colorimetric sensing method to inspection of FAs is possibly designed.

**Scheme 1:**
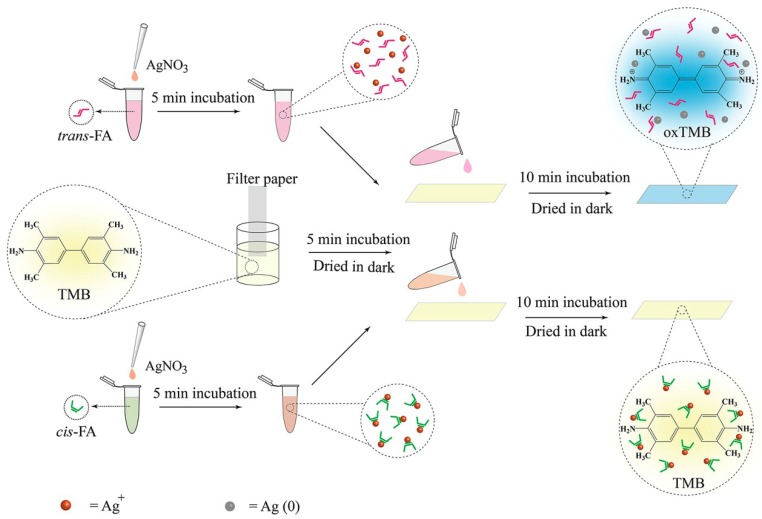
Illustration of the mechanism of test paper for colorimetric inspection of FAs according to the color changes of TMB induced by Ag^+^ oxidation.

Recently, test paper-based colorimetric sensors have been widely applicated in various fields such as disease diagnosis [23,24,25,26], environmental monitoring [27,28] and food safety inspection [29,30] due to their simplicity, disposability, economical efficiency and minimal samples requirement [31]. More importantly, they are easier to use in in situ inspections. According to the principle of paper-based colorimetric sensing, some specific reagents are necessary to develop color intensity and/or hue correlates with types and concentration of analytes [32]. Herein, considering the Ag^+^ mediated color variation of TMB and selective binding of Ag^+^ with olefinic bonds, we propose a test paper for FAs inspection (Scheme 1). By mixing Ag^+^ with FAs in a solution, a part of Ag^+^ could form Ag^+^-FAs complexes and the remnant part is free Ag^+^ ions. The free Ag^+^ could further oxidize TMB and develop blue colors. The proportion of free Ag^+^ is dependent on the concentration and type of FAs. Therefore, by taking advantage of the complexation difference of Ag^+^ to olefinic bonds of FAs, we successfully identified five types of model FAs with subtle structural differences. Using the approach described herein, the structural variation of FAs with heat treatment can be successfully monitored. Furthermore, the quality changes of mixture FAs in edible hot pot oils with heat treatment were successfully monitored by the test paper presented herein.

## 2. Materials and Methods

### 2.1. Materials

Stearic acid, oleic acid, linoleic acid, linolenic acid, elaidic acid, silver nitrate (AgNO_3_), sodium acetate and acetic acid were obtained from Aladdin Reagent Database Inc. (Shanghai, China) without further purification. TMB was purchased from Sigma-Aldrich and dissolved in ethanol with concentration of 50 mM as stock solution, further dilution was completed by using sodium acetate-acetic acid (NaAc) buffer solution. Milli-Q water (18 MΩ. cm) from a Millipore system was used in our experiments.

### 2.2. FAs Preparation

Stearic acid, oleic acid, linoleic acid, linolenic acid, elaidic acid were dissolved in ethanol with concentration of 10 mM as stock solution and diluted to different concentration for further use. For oleic acid heating experiment, oleic acid was heated at 180 °C with vigorous stirring and collected at 0, 2, 4, 6, 8 h respectively. Then the collected samples dissolved in ethanol for further colorimetric sensing. To prepare the real samples, hot pot oil was heated at 180 °C and collected at 0, 2, 4, 6, 8 h respectively. Then 1 mL of the collected oils mixed with 5 mL *n*-hexane respectively, after 10 min, the supernatant of the mixture was separated and *n*-hexane was removed with a rotary evaporator, and the remaining oils were collected for further colorimetric sensing.

### 2.3. Colorimetric Sensing of FAs

The experiments were performed in 0.2 M NaAc buffer solution with different pH range from 4.0–6.5. A 10 μL of FAs was added to 100 μL AgNO_3_ solution with final concentration range from 0.05–0.20 mM, and then 200 μL of TMB with final concentration range from 0.1–0.5 mM was added to the mixture and reacted at room temperature for 5–30 min. The UV-vis absorption spectra and digital photos of the resulting solutions were recorded on a Varian CARY 50 spectrophotometer (Agilent Technologies Inc., Santa Clara, CA, USA) and Canon 500D digital camera (Canon Inc., Tokyo, Japan) respectively.

### 2.4. Colorimetric Sensing for FAs by Test Paper

To prepare a paper-based device for FAs inspection, filter papers were cut into 0.4 cm × 2.5 cm, and immersed in TMB (0.3 mM), after 5 min incubation, filter papers were taken out, dried with nitrogen in dark and stored in a dryer without light for further use. In order to achieve FAs inspection, AgNO_3_ (0.175 mM) was firstly added in analyte FAs and incubated for 5 min; then 100 μL of the mixture was added to the as prepared paper-based device and incubated for 10 min in dark; finally, the colored papers dried with nitrogen in dark. After 10 min stabilization in dark in an ambient room, the resulting filter papers were recorded by digital camera.

## 3. Results and Discussion

**Figure 1:**
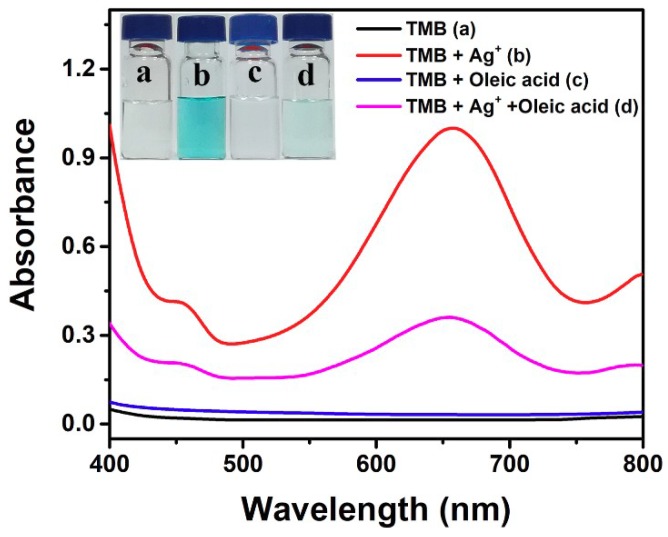
UV-vis absorption spectra of 0.3 mM TMB (**a**) without 0.175 mM Ag^+^ and 1 μM oleic acid, (**b**) with 0.175 mM Ag^+^ and without 1 μM oleic acid (**c**) with 1 μM oleic acid and without 0.175 mM Ag^+^, (**d**) with 0.175 mM Ag^+^ and 1 μM oleic acid. The inset shows the corresponding digital photo.

To demonstrate the Ag^+^-TMB sensing system could be applied to inspect of FAs, we firstly implemented some verification experiments, and the results are shown in Figure 1. The TMB alone exhibits no blue color and absorbance peaks (curve a and inset Figure 1a). When Ag^+^ was added to TMB, an obvious blue color with strong absorbance peaks centered at 652 nm was observed (curve b and inset Figure 1b), which could be attributed to the direct oxidation of TMB by Ag^+^, for there is no other reducing agent in this system. There was no blue color and absorbance peaks when oleic acid was added to TMB (curve c and inset Figure 1c), suggesting that the oleic acid alone has no effect on the oxidation of TMB. Significantly, when the mixture of oleic acid and Ag^+^ was added to TMB (curve d and inset Figure 1d), the blue color of oxidized TMB faded and its absorbance at 652 nm was decreased; because Ag^+^ complexes with the olefinic bonds of oleic acid, thus the content of free Ag^+^ is reduced and finally weakened the oxidation of TMB. According to a previous report [33], the strong complexation between Ag^+^ and FAs is attributed to the formation of σ-bonds and π-bonds between olefinic bonds and Ag^+^. Therefore, the results of Figure 1 prove the feasibility of the Ag^+^-TMB sensing system in the inspection of FAs.

**Figure 2:**
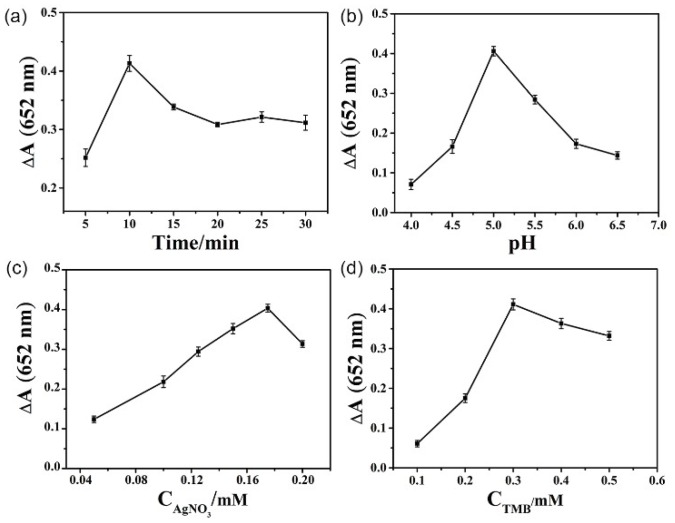
(**a**) Effect of incubation time on the Δ*A* (0.2 M NaAc buffer, pH 5.0, 0.3 mM TMB, 0.175 mM AgNO_3_ and 500 nM oleic acid). (**b**) Effect of pH on the Δ*A* (0.2 M NaAc buffer, 0.3 mM TMB, 0.175 mM AgNO_3_, 500 nM oleic acid and 10 min incubation). (**c**) Effect of AgNO_3_ concentration on the Δ*A* (0.2 M NaAc buffer, pH 5.0, 0.3 mM TMB, 500 nM oleic acid and 10 min incubation). (**d**) Effect of TMB concentration on the Δ*A* (0.2 M NaAc buffer, pH 5.0, 0.175 mM AgNO_3_, 500 nM oleic acid and 10 min incubation).

In order to realize effective colorimetric sensing, we optimized experimental conditions such as incubation time, pH, concentration of AgNO_3_ and TMB. We chose the parameter Δ*A* as a criterion for optimization; Δ*A* = *A*_0_ − *A*, *A*_0_ and *A* represent the absorbance of Ag^+^-TMB system at 652 nm in the absence and presence of oleic acid respectively. As shown in Figure 2a, the values of Δ*A* are distinct among different incubation time ranging from 5–30 min, and 10 min incubation obtained the highest value. Increasing the time has no effect on the increasing of Δ*A*, as a result, 10 min is appropriate for the experiments followed.

According to a previous report [21], the pH affected the oxidation capacity of Ag^+^, therefore we investigated the effect of pH on the Ag^+^-TMB sensing system. We adopted a 0.2 M NaAc buffer with pH range from 4.0–6.5 to determine the optimum. As shown in Figure 2b, Δ*A* increased gradually when the pH increased from 4.0 to 5.0, and when the pH was greater than 5.0 the Δ*A* decreased gradually, therefore, we chose pH 5.0 for the FAs sensing experiments.

Finally, the concentration of TMB and AgNO_3_ were optimized. As shown in Figure 2c,d, the too high or too low concentration of TMB and AgNO_3_ are not appropriate for FAs sensing. On the one hand, the higher concentration of TMB and AgNO_3_ needs higher concentrations of FAs to produce the recognizable color changes, which reduces the sensitivity of the sensing system. On the other hand, the low concentration of TMB and AgNO_3_ makes the low absorbance of oxidized TMB which introduces the larger experimental error. When the concentration of TMB and AgNO_3_ are 0.3 mM and 0.175 mM, the highest Δ*A* are obtained respectively. Therefore, we chose 0.3 mM of TMB and 0.175 mM of AgNO_3_ for the next FAs sensing experiments.

**Figure 3:**
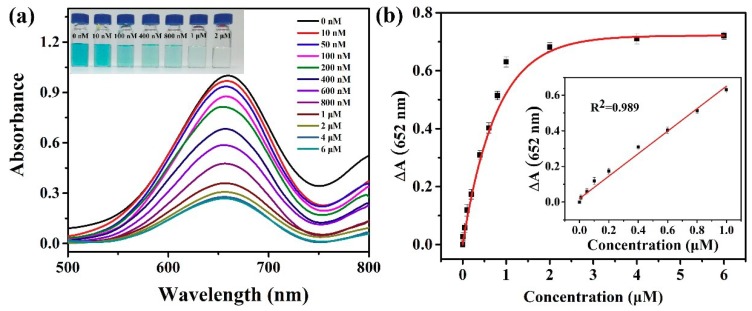
(**a**) Absorption spectra (inset is digital photo) of Ag^+^-TMB system in the presence of different concentrations of oleic acid (0 to 6 μM, from top to bottom); (**b**) Plots of Δ*A* versus different concentrations of oleic acid ranging from 0 to 6 μM (inset is the linear relationship between Δ*A* and the concentration of oleic acid from 0 to 1 μM). Experimental conditions: 0.2 M NaAc buffer (pH = 5.0), 0.3 mM of TMB and 0.175 mM of AgNO_3_, incubation time is 10 min. The error bars represented standard deviations based on three independent measurements.

To verify the sensitivity and concentration-dependent response of Ag^+^-TMB system for FAs, the effect of 0 to 6 μM oleic acid on the color change of Ag^+^-TMB system was investigated. As shown in the inset of Figure 3a, under the optimal experimental conditions, the color changes of the Ag^+^-TMB system induced by the variation of oleic acid concentration can be clearly visualized. The dark blue color of Ag^+^-TMB system is gradually weakened to light blue with the increasing of oleic acid concentration. Meanwhile, the corresponding absorbance of Ag^+^-TMB system at 652 nm is decreasing (Figure 3a) as the oleic acid concentration is increasing. Correspondingly, Δ*A* with the increasing concentration of oleic acid were investigated (Figure 3b). It is obviously seen that Δ*A* increased gradually with the increasing of oleic acid concentration, and there is no obvious increase observed when the concentration of oleic acid is up to 2 μM. In the range of 0–1 μM, Δ*A* increased linearly with increasing oleic acid concentration (inset of Figure 3b), and the equation fitted as Δ*A* = 0.01 + 0.63*C* (*C* is the concentration of oleic acid), with the corresponding R^2^ = 0.989.

**Figure 4:**
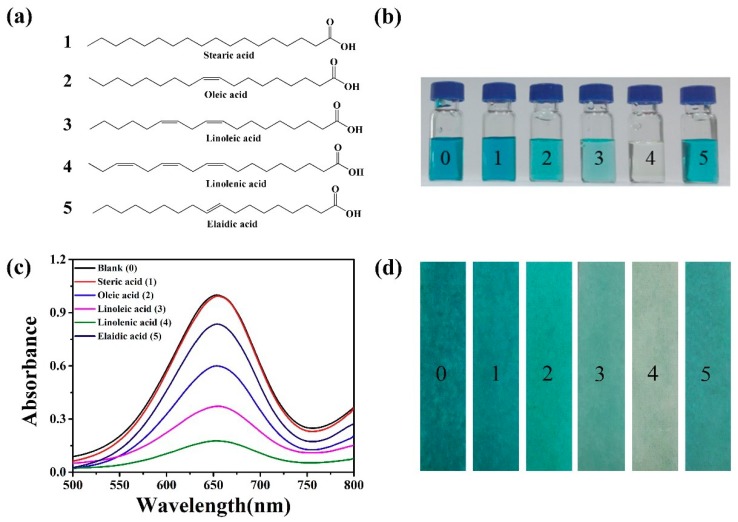
(**a**) Molecular structures of five FAs; (**b**) digital photo and (**c**) absorption spectra of Ag^+^-TMB sensing system towards five FAs (FAs concentration: 500 nM); (**d**) test papers towards five FAs. The meaning of numbers: 0 (blank), 1 (steric acid), 2 (oleic acid), 3 (linoleic acid), 4 (linolenic acid), 5 (elaidic acid).

In order to demonstrate the selectivity of the Ag^+^-TMB system, five FAs including stearic acid (SFAs), oleic acid (mono-CUFAs), linoleic acid (poly-CUFAs), linolenic acid (poly-CUFAs) and elaidic acid (TUFAs) with different unsaturation degrees and configuration of the olefinic bonds (Figure 4a) were investigated. Figure 4b is the digital photo of Ag^+^-TMB sensing system towards the above five FAs, and Figure 4c is the corresponding absorption spectra. The number of 0 (blank) represents Ag^+^-TMB system with the addition of equal volume of ethanol without any FAs addition as the control experiment, and numbers 1–5 represent the above five FAs respectively. As shown in Figure 4b, five FAs displayed distinct colors ranging from dark blue to colorless, and the five types of FAs were readily distinguished from blank by the naked eye except for stearic acid (1). Because stearic acid has no olefinic bond, it cannot complex with Ag^+^ and produce color change. A similar result is observed in Figure 4c, that is, the addition of stearic acid has almost no effect on the change of absorption spectrum of Ag^+^-TMB system. For oleic acid (2), linoleic acid (3) and linolenic acid (4), they are all CUFAs but with different numbers of olefinic bonds: 1, 2 and 3 respectively. With the number of olefinic bonds increasing, the color changed from dark blue to colorless, and the corresponding absorbance of Ag^+^-TMB system at 652 nm gradually decreased. The reason for this is that the complexation ability of FAs with Ag^+^ increased with the number of olefinic bond increasing. Linolenic acid shows the lowest absorption at 652 nm and almost colorless, for it has 3 olefinic bonds and complexes with Ag^+^ more easily than other two FAs. As for different configurations of FAs, for example, in the case of oleic acid (2) and elaidic acid (5) which only have one olefinic bond at same position but with different configurations, the result of colorimetric sensing is distinct. Elaidic acid exhibits darker blue and higher absorption at 652 nm compared with oleic acid. Because the complexation between Ag^+^ and *trans*-olefinic bond is weaker than *cis*-configuration, therefore, the degree of TMB oxidation caused by *trans*- is more acute than *cis*-configuration. As a result, the color of Ag^+^-TMB system in the presence of oleic acid is lighter than the presence of elaidic acid.

Next, we designed a test paper for colorimetric sensing of FAs based on the proposed Ag^+^-TMB sensing system to improve the portability and disposability of this sensing system. As shown in Figure 4d, the paper-based devices exhibit distinct colors toward the above five types of FAs and could be easily distinguished from blank (0) except for steric acid (1). CUFAs including oleic acid (2), linoleic acid (3) and linolenic acid (4) shows the gradual fading blue color for their number of olefinic bond are increasing. As for configurational identification, the elaidic acid (5) shows darker blue than oleic acid (2), for TUFAs has lower ability to complex with Ag^+^ than CUFAs. Thus, the results indicate that the delicately designed test paper shows good selectivity for different types of FAs; and it’s worth noting that the preparation processes of test paper were very simple, which could be widely used in in situ inspection of FAs.

**Figure 5:**
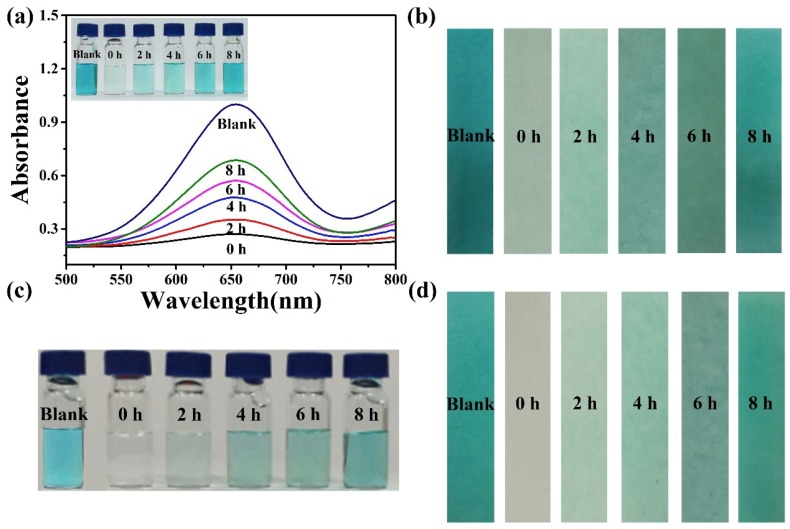
(**a**) Absorption spectra (inset is digital photo) of Ag^+^-TMB sensing system and (**b**) test papers toward oleic acid samples with heat treatment at 180 °C for different times; (**c**) digital photo of Ag^+^-TMB sensing system and (**d**) test papers toward hot pot oils with heat treatment at 180 °C for different time.

Furthermore, the test paper could be used in monitoring of FAs structural variations, and we took oleic acid as example to illustrate. According to a previous report [34], heating could induce FAs variation in terms of unsaturation degree and generation of harmful *trans*-configurations; thus, we implemented experiments to monitor the structural variation of oleic acid by the proposed approach. As shown in the inset of Figure 5a, oleic acid without heating (0 h) exhibits a very light blue color, while when the heating time increased from 0 to 8 h, the color of Ag^+^-TMB solution is gradually deepened to dark blue and the absorption of the sensing system at 652 nm is gradually increased. Correspondingly, the test paper shows the similar results (Figure 5b). The color changes of the sensor are ascribed to the unsaturation and configuration variation of oleic acid induced by heating.

In order to prove the feasibility of the test paper for the in situ inspection of complex mixture FAs, we profiled the quality changes of edible hot pot oils with heat treatment based on the method described herein. Edible oils are a very important source of FAs, and usually suffered from variations of unsaturation degree and configuration, which occurs when fresh edible oils are heated at high temperatures during various food preparations [35]. Illegal gutter oils are refined second-hand oils which is detrimental to people’s health because their quality have been declined, but it is common to use these oils in the commercial food industry for maximized profits. Therefore, the inspection of the quality of edible oils is significant. As a very famous special food in China, hot pot is very popular there, and the oils used for this cuisine suffered continuous heating during the process of eating. There are many reported food safety incidents regarding second-hand oils being used in hot pot, therefore the quality monitoring of the hot pot oils is very important. Soybean oil is a commonly used edible oil in hot pot, which mainly composed of CFAs (ca. 81%), with saturated FAs as minor components (ca. 18%) [36]. In our work, we monitored the quality changes of soybean oil under continuous heating with different times. As shown in Figure 5c, the original oil (0 h) without heating is almost colorless, and with the increased heating time from 0 to 8 h, the color of the sensing system gradually turns dark blue. In the paper-based sensing results (Figure 5d), the original oil (0 h) shows the color of filter paper, and the color of paper gradually changed to dark blue with as the heating time increased. The color changes were mainly attributed to the variation of unsaturation degree and configuration of FAs, which lower the complexation ability of hot pot oil with Ag^+^, thus the adequate free Ag^+^ could oxidize TMB and develop blue colors. These results indicate that the portable and disposable test paper can be used to monitor the changes in oil quality by the naked eye, and the method can be used in in situ safety inspection of edible oils.

## 4. Conclusions

In this work, we proposed a test paper for the colorimetric inspection of FAs with subtle difference in their alkyl chains such as number, position and configuration of olefinic bonds. For the first time, the approach we designed herein could discriminate FAs rely on the highly selective binding of Ag^+^ to olefinic bonds in FAs rather than the shape of alkyl chain (bent or linear). Based on the Ag^+^ mediated color variation of TMB, the test paper achieved colorimetric discrimination of five types of model FAs including stearic acid, oleic acid, linoleic acid, linolenic acid, and elaidic acid. Furthermore, the test paper was successfully applied to monitor the structural variations of oleic acid with heat treatment. Significantly, the quality changes of mixture edible hot pot oils with heat treatment was successfully monitored by the sensor. Thus, the facile, economic, portable and disposable test paper for FAs holds great potential in in situ food safety inspection.
